# Choroidal eye metastases from (recurrent) primary peritoneal carcinoma: case report and review of the literature

**DOI:** 10.1186/1477-7800-6-3

**Published:** 2009-01-20

**Authors:** Nikolaos Thomakos, Khandra Galaal, Georgios Georgopoulos, Lakshmi Nagaraju, Dianne Hemming, Raj Naik

**Affiliations:** 1 Northern Gynaecological Oncology Centre, Gateshead Health NHS Trust, Queen Elizabeth Hospital, Sheriff Hill Gateshead NE9 6SX, UK; 2 Department of Pathology, Gateshead Health NHS Trust, Queen Elizabeth Hospital, Sheriff Hill Gateshead NE9 6SX, UK; 3 Ophthalmology Department, Social Insurance Institute, Athens, Greece

## Abstract

**Background:**

Choroidal metastases from gynaecological primary are extremely rare. There is no documented case in the literature of choroid metastasis in a patient with primary peritoneal carcinoma (PPC).

**Methods & Results:**

We describe the first case of a 54-year-old woman with a history of borderline mucinous tumour who presented 17 months later with PPC and 21 months after with recurrent disease metastatic to the eye, and review pertinent literature.

**Conclusion:**

High index of suspicion is warranted when patients with history of primary peritoneal carcinoma present with visual complaints in order to treat and/or relieve symptomatology from metastatic eye disease.

## Background

Primary peritoneal carcinoma (PPC) is a rare tumour that is histologically similar to primary epithelial ovarian carcinoma (EOC). Its diagnosis is based on the extent of gross ovarian involvement and microscopic invasion of the ovarian cortex. It diffusely involves the peritoneal cavity often with extensive disease in the upper abdomen affecting mostly the omentum, while sparing or minimally involving the ovaries with microscopic or small macroscopic cancer deposits on the surface of the ovary [1].

It has been shown with the use of histopathologic criteria that these tumors appear more like Mullerian neoplasms than classic mesotheliomas [2].

Since PPC has the appearance of a 'Mullerian' carcinoma and simulates ovarian cancer clinically, distant metastases may occur in the pleura, lung, liver and lymph nodes, while the central nervous system (CNS) remains one of the uncommon sites of metastases [3,4]. Moreover, eye involvement from such malignancies is exceedingly rare, with only seven cases of epithelial ovarian cancer being reported in the literature [5-9].

There is no documented case in the literature of choroid metastasis in a patient with primary peritoneal carcinoma (PPC). This case represents the first documented report of choroidal eye metastasis from PPC. We also review briefly the related literature using a Medline search and discuss our findings in the present case.

## Case presentation

A 54-year-old Caucasian woman presented with abdominal distension and on clinical examination was found to have a large ovarian cyst extending up to the xiphisternum. Abdomino-pelvic ultrasound confirmed a multi-loculated cyst. Nineteen years previously she had undergone total abdominal hysterectomy and left salpingo-oopherectomy for endometriosis. The CA 125 level was within the normal limits at 25 u/ml. Laparotomy, washings, right salpingo-oophorectomy and omentectomy was performed. Histology showed a borderline mucinous tumour and the peritoneal washings showed reactive mesothelial cells, FIGO stage 1A. After the multidisciplinary team meeting, no further treatment was recommended. The CA 125 level reverted to normal and she was discharged from hospital follow-up in August 2004.

Four months later she presented with right-sided upper abdominal pain. CA125 was found to be raised 2036 u/ml. Computed tomography (CT) of the abdomen and pelvis revealed ascites with disseminated peritoneal deposits and a solitary left lower lobe lung metastasis, but no evidence of malignant disease in the upper abdomen including pancreas. Diagnostic laparoscopy and biopsies were performed; the findings were those of miliary peritoneal and diaphragmatic disease and an omental cake adherent to anterior abdominal wall. The histology of the biopsy from the diaphragm and peritoneum revealed a poorly differentiated adenocarcinoma consistent with primary peritoneal carcinoma. The ascitic fluid was positive for malignant cells.

**Figure 1:**
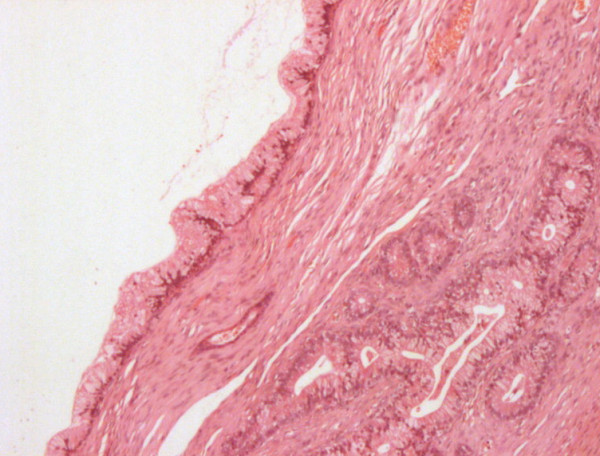
**Borderline mucinous tumour 269 × 204 mm (72 × 72 DPI)**.

The previous pathology including that of the right ovary from the first laparotomy were reviewed. The patient was confirmed to have had a borderline mucinous tumour of the right ovary with the tumour cells staining positive for cytokeratin 7 (CK7) and CEA but negative for CA 125 and cytokeratin 20 (CK 20) (Figure 1).

**Figure 2:**
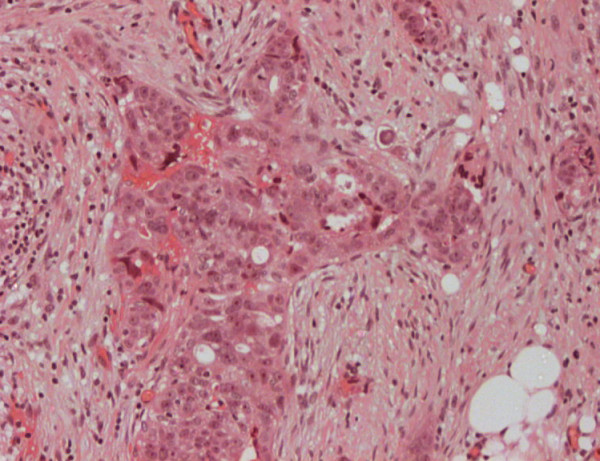
**Primary peritoneal carcinoma 269 × 204 mm (72 × 72 DPI)**.

The laparoscopic biopsies taken from the subsequent disseminated peritoneal malignancy showed poorly differentiated adenocarcinoma consistent with primary peritoneal carcinoma (possible endometrioid type)and showing a different immunostaining pattern to the previous ovarian borderline mucinous tumour with positive expression of cytokeratin 7(CK7) and CA 125 but negative for CEA and CK 20. (Figure 2).

Subsequently the patient received three cycles of neoadjuvant chemotherapy with carboplatin and paclitaxel followed by interval debulking surgery that revealed two areas of disease in the omentum, which were excised. The end result was optimal cytoreduction with no intra-abdominal macroscopic disease. The final histology confirmed poorly differentiated adenocarcinoma. Post-operatively a further three cycles of chemotherapy was given with the CA 125 level normalised to 22 u/ml by the end of treatment.

Four months later, the patient again presented with raised CA 125 at 198 u/ml and pain under her left lower ribs. Computed tomography of abdomen and pelvis showed tumour recurrence in the abdomen with pulmonary and liver metastases. The patient was then started on second line chemotherapy with liposomal doxorubicin.

After 8 cycles of liposomal doxorubicin a CT scan of the abdomen and pelvis showed enlarged liver metastases with evidence of progressive disease. The CA 125 level had further increased to 724 u/ml.

**Figure 3:**
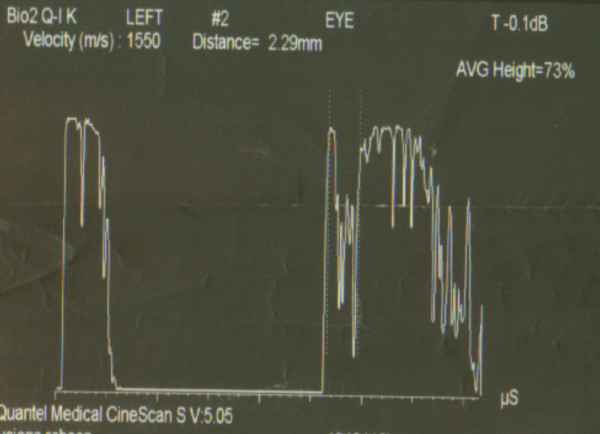
**A-Scan, Amplitude modulator 301 × 203 mm (150 × 150 DPI)**.

**Figure 4:**
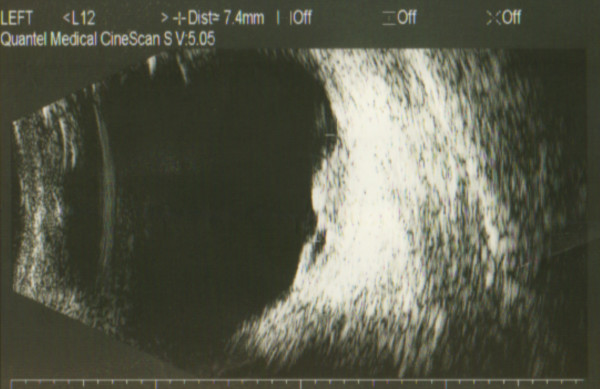
**B-Scan, Brightness modulator 326 × 203 mm (150 × 150 DPI)**.

One month later, she presented with one-week history of blurring of vision in the left eye. She was referred for ophthalmologic evaluation, where the bilateral anterior segments, the pupils and lenses including the fundus of the right eye were found to be normal. However, fundoscopy of the left eye revealed a creamy dome shaped mass in the superior part of the left fundus. Further investigations with diagnostic ultrasonography of the ocular structures were then performed. The patient underwent A (amplitude modulation) and B (brightness modulation) ultrasound scan of the eye (the A-scan usually reveals medium to high internal reflectivity while the B-scan will show acoustic solidity) [10]. The A scan showed medium to high internal reflectivity irregular maximum elevation of 2.29 mm (figure 3), while the B scan showed a dome shaped lesion in the superior part of left fundus with surrounding elevation of the retina (figure 4). The ophthalmoscopic and ultrasound investigations were consistent with choroidal metastasis. The patient subsequently received palliative radiotherapy of 20 Gy in 5 fractions following which her symptoms of blurred vision and pain in the left eye improved.

The patient's condition steadily deteriorated from then on with pain in the right hypochondrium, right shoulder and right posterior chest associated with breathlessness. Computed tomography pulmonary angiography (CTPA) revealed left lower lung metastases with mediastinal encasement by metastatic disease with secondary pericardial effusion and a moderate left pleural effusion and small pulmonary emboli. The pleural and pericardial effusions were drained to control symptoms. The cytology of the pericardial fluid confirmed the presence of malignant cells. She subsequently developed refractory renal failure and died.

## Conclusion

The incidence of metastatic carcinoma to the eye varies by reports and ranges from 10% – 12.6% in autopsy studies of patients with disseminated cancer [11,12] to 0.07% – 2.3% in patients with advanced generalized malignancy [13]. The commonnest primary site is breast cancer in 47–49% of cases and lung cancer in 14–21% of cases [14,15]. The choroid is the most common site in the eyes, with uveal metastases (88%) whereas 9% locate in the iris and 2% in the ciliary body. The tumours occur most often in the posterior pole of the eye with an average of two focuses per eye [14].

Ovarian cancer metastatic to the central nervous system (CNS) is reported to occur in less than 1.5% of all ovarian cancer metastases [4,16]. The incidence of CNS metastases in patients with ovarian cancer is reported to range from 0.29% in a review of 4456 patients [17] to 1.2% in an autopsy study of 86 patients [18].

Choroidal involvement from gynaecological malignancies is extremely rare. A review of the literature found only seven cases of choroidal involvement from ovarian carcinoma [5-9,19,20] four cases from cervical cancer [13,21,22] and one case of endometrial adenocarcinoma [23]. The choroidal metastases are usually diagnosed on fundoscopy, while imaging techniques including ultrasound examination of the eye, CT scan of the head and orbit and MRI scanning are used as well. Fluorescein angiography may be helpful showing early hyperfluorescence and diffuse late staining of the lesion [9,19,20,23,24]. In our case the patient had disseminated primary peritoneal carcinoma with evidence of systemic recurrence in the liver and lungs and therefore the eye metastasis was deemed to originate from disseminated primary peritoneal carcinoma.

We believe that the present case is the first reported case of PPC with metastasis to the choroid of the eye. Moreover, this patient is unique in that the choroidal metastases occurred within three years of having a borderline mucinous tumour of the ovary and primary peritoneal carcinoma. In respect to the patient's history of endometriosis it is possible that the disseminated peritoneal tumour may have originated from endometriosis of the peritoneal surfaces. It has been reported that 69% of the malignancy arising from extra-gonadal sites of endometriosis including the pelvic peritoneum show endometrioid differentiation [25].

Although the primary mode of dissemination for EOC and PPC is by transcoelomic spread, haematogenous spread can occur. It seems that the high vascularity of the uvea and specifically of the choroid predisposes the eye to intraocular metastases. The average survival time of patients with choroidal metastasis from any primary carcinoma is 8–9 months after diagnosis [26]. Review of the reported cases showed that the mean duration from diagnosis of EOC to detection of choroidal metastases was 23 months and the mean duration from diagnosis of metastases to death was 8 months [5-9,19,20]. Our patient's choroidal eye metastases appeared 21 months after PPC was diagnosed

Although prognosis is poor, treatment of the eyes should be considered in order to preserve the visual function and/or relieve pain. Local radiotherapy has been shown to be an effective approach and provides significant palliation of symptoms [10], whereas multimodal treatment approach including systemic chemotherapy and local radiotherapy appears to be another option. Transpupillary thermotherapy (TTT) with diode laser can be used for deposits of medium thickness and minimal amount of subretinal fluid [24]. Response to treatment has been reported to be about 81% with remission of ocular disease in 59% [27]. In cases of painful blind eye, enucleation may be considered [24].

Since the incidence of distant metastasis from PPC appears to be increasing [6], we believe that both the earlier recognition of the disease and the use of effective chemotherapeutic regimens might offer patients significant local control and long-term survival benefit allowing occult metastatic disease to be picked up and therefore treated earlier. Although metastases to the eyes are unusual, this case demonstrates the need for suspicion and prompt investigation of patients with visual complaints and history of primary peritoneal carcinoma.

## Competing interests

The authors declare that they have no competing interests.

## Authors' contributions

NT participated in the design of the case report, KG participated in the design of the literature review, GG participated by adding further ophthalmological infical analysis, RN participated in the design and coordination of the manuscript. All authors read and approved the final manuscript.

## Consent

Since the patient has died, consent was not obtainable, but the relatives has been informed, and are in agreement with the intention to write and publish this paper.
